# Examining the Limits of Predictability of Human Mobility

**DOI:** 10.3390/e21040432

**Published:** 2019-04-24

**Authors:** Vaibhav Kulkarni, Abhijit Mahalunkar, Benoit Garbinato, John D. Kelleher

**Affiliations:** 1 Department of Information Systems, UNIL-HEC Lausanne, 1015 Lausanne, Switzerland; 2 Applied Intelligence Research Center, Technological University Dublin, D08 NF82 Dublin, Ireland

**Keywords:** mobility predictability limits, entropy convergence, mutual information, mobility modeling

## Abstract

We challenge the upper bound of human-mobility predictability that is widely used to corroborate the accuracy of mobility prediction models. We observe that extensions of recurrent-neural network architectures achieve significantly higher prediction accuracy, surpassing this upper bound. Given this discrepancy, the central objective of our work is to show that the methodology behind the estimation of the predictability upper bound is erroneous and identify the reasons behind this discrepancy. In order to explain this anomaly, we shed light on several underlying assumptions that have contributed to this bias. In particular, we highlight the consequences of the assumed Markovian nature of human-mobility on deriving this upper bound on maximum mobility predictability. By using several statistical tests on three real-world mobility datasets, we show that human mobility exhibits scale-invariant long-distance dependencies, contrasting with the initial Markovian assumption. We show that this assumption of exponential decay of information in mobility trajectories, coupled with the inadequate usage of encoding techniques results in entropy inflation, consequently lowering the upper bound on predictability. We highlight that the current upper bound computation methodology based on Fano’s inequality tends to overlook the presence of long-range structural correlations inherent to mobility behaviors and we demonstrate its significance using an alternate encoding scheme. We further show the manifestation of not accounting for these dependencies by probing the mutual information decay in mobility trajectories. We expose the systematic bias that culminates into an inaccurate upper bound and further explain as to why the recurrent-neural architectures, designed to handle long-range structural correlations, surpass this upper limit on human mobility predictability.

## 1. Introduction

The proliferation of mobile devices equipped with internet connectivity and positioning systems has resulted in the collection of large amounts of human-mobility data. Real-time user locations are typically collected using the global positioning system (GPS), call detail record logs (CDR) and wireless-LAN (WLAN). The resulting location datasets can be then used to study and model user mobility behaviors, beneficial to a variety of applications, such as traffic management, urban planning and location-based advertisements. One of the applications of mobility modeling consists of formulating predictive models to forecast individual trajectories. For this, various methods have been proposed, including Markov chains [1], neural networks [2] and finite automata [3]. Existing research has used several datasets differing with respect to their spatial and temporal granularity, resulting in vastly contrasting prediction accuracies ranging from over 90% to under 40% [4].

### 1.1. Benchmarking Limits of Mobility Prediction

In this context, the seminal paper of Song et al. [5] laid the foundations for computing a theoretical upper bound on the maximum predictability of human mobility. This work establishes a benchmark for quantifying the performance of different prediction models and generalizes its approach across various datasets. The goal of mobility prediction is to predict the next visited user location with the highest possible accuracy, quantified in terms of the proportion of accurate predictions, noted as $\pi_{acc}$. Song et al. [5] define predictability upper bound, noted $\pi^{max}$, as the highest potential accuracy of modeling the mobility behavior of individuals present in a given dataset (highest possible $\pi_{acc}$). The value of $\pi^{max}$ is defined by the entropic level of this dataset, and lower entropy would imply higher predictability. The derived $\pi^{max}$ is experimentally corroborated by constructing a prediction model and computing $\pi_{acc}$, accuracy of forecasting user locations on the same dataset. Given that $\pi^{max}$ is the upper bound of prediction accuracy as defined by Song et al. [5], $\pi_{acc}\leq \pi^{max}$ should always hold.

We highlight that $\pi^{max}$ should not be confused with *predictability horizon* [6], which is defined as the limit of how far ahead one can predict (utmost prediction range), given a mobility dataset. The question therefore is not how long is the horizon of the predictability limit, but given a horizon (the next time instance in this case) what is the maximum possible predictability. The prediction model will contain some amount of uncertainty within this horizon which is limited by the chaotic nature of the individuals’ mobility behavior present in the dataset. Furthermore, the computation of $\pi^{max}$ is dependent exclusively on the mobility patterns of individuals and does not account for any supplementary information. To this end, Qin et al. [7] estimate the maximum predictability given a single location instance and quantify how predictable individuals are in their mobility.

In practice, Song et al. [5] compute $\pi^{max}$ by first estimating the entropy of the mobility trajectories contained in the dataset based on Lempel–Ziv data compression [8]. This entropy estimate is used to solve the limiting case for Fano’s inequality [9]. Fano’s inequality [9] is an information-theoretical result used to compute lower bound on the minimum error probability in multiple-hypotheses testing problems. The estimated lower bound is then used to compute the maximum possible accuracy of predictability ($\pi^{max}$). The proposed theoretical upper bound by [5] ($\pi^{max}=93\%$) is computed using a call detail record (CDR) dataset consisting of 50,000 users collected by a telecommunications operator for a duration of three months. They also show that $\pi^{max}$ is independent of radius of gyration and movement periodicity, hence they observe an insignificant level of variation across a heterogeneous population.

Several subsequent works computed $\pi^{max}$ using datasets of different types, collected for varying durations and performed empirical validation by constructing Markov based prediction models [10]. Lu et al. [11] estimate $\pi^{max}$ to be 88% for a call detail record (CDR) dataset consisting of 500,000 users, collected for a duration of five months. In order to validate this bound, they use *order-1* Markov chain based prediction model and achieve an average prediction accuracy ($\pi_{acc}$) of 91%. They also show that higher-order Markov chain models do not significantly improve the prediction accuracy. Their interpretation behind surpassing their own estimated theoretical bound is that trajectories exceeding this bound are non-stationary, whereas the accuracy of stationary trajectories prevails within the bound. A trajectory is considered to be stationary when people tend to remain still during short time-spans. This conclusion directly contradicts findings of Song et al. [5], because non-stationary trajectories should by definition have a higher entropy. Later, Cuttone et al. [4] show that the stationary nature of trajectories plays a significant role in the higher accuracies resulting from Markov models [4] as they often predict the user will remain in the previous location, i.e., self-transitions. Lin et al. [1] also show that $\pi^{max}$ is independent of the spatial granularity (Δt) data sampling rate (Δt) which was later questioned by Smith et al. [12] ($\pi^{max}=93\%-74\%$ for varying values of Δs and Δt) and Cuttone et al. [4] ($\pi^{max}=65\%$). Smith et al. [12] and Cuttone et al. [4] used mobility datasets [13,14] containing GPS trajectories and showed that predictability has a direct correlation with the temporal resolution and an inverse correlation with the spatial resolution.

The CDR datasets used in the preliminary works [5,11] are known to have inherent gaps due to the short bursts of calls masking the user’s true entropy. Therefore, it should be noted that CDRs are a rough approximation of human mobility due to the low granularity of GSM cell IDs. Since human mobility varies under time translations, the entropy not only depends on the duration of past observations but also on number of visited locations; these factors tend to be hidden in such datasets [15,16]. Additional inconsistencies become evident due to the fact that the authors in [5,11] group the user locations into one-hour bins when constructing the historical trajectory of a user. Further inspection suggests that these models can foresee future locations at $\pi^{max}$, only when an individual is present in one of the top *n* bins [4]. The first two works [5,11] thus consider the last location of each day, consequently predicting only the user’s home place. Under such a scenario, Ikanovic et al. [17] and Cuttone et al. [4] showed that the predictability of the true next location is significantly lower ($\pi^{max}=71.1\pm 4.7\%$) than the predictability of the location in the subsequent bin. They further showed that an individual’s mobility entropy is directly proportional to the number of visited locations. The authors also point out that the generating function behind the stochastic mobility behavior is often unknown. Therefore the bounds cannot be estimated theoretically and require empirical derivation. Cuttone et al. [4] achieve an even lower bound on $\pi^{max}$ of 65% on the same datasets with the same methods as Ikanovic et al. [17].

In this paper, we build upon the work of Zhao et al. [18], who demonstrate the non-Markovian character of the online and offline human behavior. They analyze datasets consisting of user web browsing and location-visit patterns and estimate the rank distribution of these visits. They show the presence of the scaling law associated with the dwelling times at the visited websites and locations. This study hints at the non-Markovian character and is based on a small scale CDR dataset using one-point statistics [19]. However, mobility trajectories involve complex dynamics, which are better characterized by two-point statistics [1], hence the work of Zhao et al. [18] is inconclusive. On the contrary, we study the mobility characteristics through the lens of both these methodologies on three large scale datasets collected at varying levels of spatiotemporal granularities. This minimizes any bias and substantiates our findings.

**Problem Definition.** Before going into further details, we can state the central objective of this work as follows: knowing that we observe a discrepancy between the predictability upper bound and the empirical prediction accuracy, we aim at investigating the methodology behind the upper bound estimation and at understanding the primary reasons for this discrepancy. To this end, we adopt the approach consisting in the three steps listed hereafter, where each step acts as a causal verification for the next.

Approach:

1. Confirm the discrepancy between the upper limit of predictability and prediction accuracy through extensive experimentation using widely contrasting prediction models on contrasting datasets.
2. Following the discrepancy confirmation, revisit the assumptions underlying the upper bound computation methodology.
3. Scrutinize the assumptions, analyze the reasons contributing to the failure of the methodology.

### 1.2. Discrepancies and Inconsistencies

Table 1 summarizes the findings of previous works by indicating the $\pi^{max}$ values, the prediction accuracy score ($\pi_{acc}$), prediction model used and the type/duration of the dataset used. As seen in the Table 1, ref [5] and [12] do not compute the $\pi_{acc}$ scores, while [11] estimates $\pi_{acc}>\pi^{max}$, contradicting findings of [5]. We also observe that $\pi^{max}$ is impacted by Δs and Δt as evident from the work of [12] and [17], contracting [5] and [11]. Therefore, we observe an inconsistency regarding the maximum predictability bound $\pi^{max}$ and its relation to $\pi_{acc}$. We also observe disagreements regarding the impact of entropy, on the number of uniquely visited locations and on the spatiotemporal resolution of the trajectory on $\pi^{max}$. Moreover, the $\pi_{acc}$ derived by some works [11,17] based on Markov chains surpass the limits of their own $\pi^{max}$. To systematically revisit the above discrepancies and inconsistencies, we compute the values of $\pi^{max}$ for three large scale mobility datasets and estimate $\pi_{acc}$ using seven different prediction techniques for benchmark and comparison purpose.

### 1.3. Questioning the Predictability Upper Bound

In this paper, we challenge the validity of the currently established mobility predictability upper bound following our own observation that recurrent-neural networks surpass this limit. Our central objective is a comprehensive inspection of the methodology behind the derivation of this upper limit and identify the probable causes behind this anomaly. This involves analyzing and confirming the two phenomenon described hereafter.

1. Substantiate the observed discrepancy between $\pi_{acc}$ and $\pi^{max}$. To this end, we build prediction models using seven distinct approaches and conduct a comprehensive accuracy analysis based on three real-world mobility datasets.
2. Revisit the assumptions hereafter, which might have lead to this discrepancy.
  (a) Human mobility is Markovian and thus possesses a memoryless structure.
  (b) The mobility entropy estimating technique achieves an asymptotic convergence.
  (c) The predictability upper bound accounts for (all) the long-distance dependencies in a mobility trajectory.

### 1.4. Roadmap and Main Findings

We now sketch the relevant contributions and present the organization of this paper.

1. We discuss all the relevant concepts used in this work in Section 2 and illustrate how diverse concepts such as entropy, mutual information and predictive information interact with each other in the light of the predictability upper bound.
2. In Section 3, we describe the mobility datasets used in this work and confirm the discrepancy between the maximum upper bound of mobility prediction derived by the previous works and the empirical prediction accuracy derived using recurrent-neural network variants. In order to minimize any bias, we construct seven different prediction models and compute the accuracy across three datasets differing with respect to their collection timespans, region, demographics, sampling frequency and several other parameters.
3. In Section 4, we audit three underlying assumptions in the currently used methodology for $\pi^{max}$ computation.
  (a) In Section 4.1, we demonstrate the non-Markovian character of human mobility dynamics contrary to the previously held assumption. Our statistical tests to confirm the nature of human mobility include (i) rank-order distribution, (ii) inter-event and dwell time distribution, and (iii) mutual information decay.
  (b) In Section 4.2, we analyze the entropy convergence by comparing entropies derived by using Lempel–Ziv 78 and Lempel–Ziv 77 encoding schemes on mobility trajectories. Based on this result, we show that there does not exist an ideal entropy estimation scheme for mobility trajectories that achieves an asymptotic convergence.
  (c) In Section 4.3, we assert that the current methodology used to estimate $S^{real}$ does not represent an accurate entropy estimate of mobility trajectory. To this end, we demonstrate that the individual elements present in a mobility subsequence derived by the currently used encoding schemes have non-zero dependencies un-accounted for, when deriving the mobility entropy. We validate such a manifestation by computing the pointwise mutual information associated with mobility trajectories which indicate an on average positive pointwise mutual information (PMI).
4. In Section 5 we discuss the likely causes behind this discrepancy being overlooked. We also present the potential reasons as to why recurrent neural networks (RNN) extensions exceed the theoretical upper bound and discuss the applicability of the prediction models in different contexts. We conclude the paper in Section 6.

## 2. Relevant Concepts

In this section, we present the key concepts and principles relied upon in this work. We present a brief description of Markov processes which have been widely used in human mobility prediction, followed by the definition of long-distance dependency (LDD). We further relate the discussion regarding long-distance dependencies (LDDs) with their quantification through mutual information. We then present the relationship between entropy, encoding and data compression, followed by the conceptual understanding of predictability theory and its relation to the above concepts.

### 2.1. Mobility Modeling

A mobility modeling task aims at estimating the probability distribution over a user’s location traces by minimizing the negative log-likelihood of the training sequences [20]:

(1) $$min\theta -\frac{1}{N}\sum_{n=1}^{N}\sum_{t=1}^{T^{n}}\log p\left(L_{t}^{n}|L_{<t}^{n};\theta \right),$$

where θ is the model parameter, *N* is the number of training location traces, and $T^{n}$ is the length of the *n*-th location trace. A location or a symbol at time *t* of trace *n* is denoted by $L_{t}^{n}$, and $L_{<t}^{n}$ denotes all the previous locations (symbols) at time *t*.

### 2.2. Markov Processes

Several of the previous works related to human mobility modeling [10], mobility prediction [21,22] and derivation of the upper bound [5,11] rely on the Markov assumption of human mobility. Markov processes are a natural stochastic extension of finite state automata, where the state transitions are probabilistic and there is no input to the system in contrast to a finite state automaton. Thus, the observation at a given time $t_{i}$ only depends on events at previous time step $t_{i-1}$ or on previous *n* time steps for an *n*-order Markov chain. Such stochastic processes are characterized in terms of the transition probabilities, where the probability for transitioning to the next state is an exponentially distributed random variable. Formally, a sequence of random variables $X_{1},X_{2},X_{3},\ldots$ abide the Markov property as expressed in Equation (2).

(2) $$P\left(X_{n}=x|X_{n-1}=x_{n-1},\ldots ,X1=x_{1}\right)=P\left(X_{n}=x|X_{n-1}=x_{n-1}\right),$$

where P(A|B) is the probability of *A* given *B*. The possible values of $X_{i}$ form a countable set called the state space of the Markov chain.

### 2.3. Long-Distance Dependencies

A long distance dependency (LDD) describes a contingency or interaction between two or more elements in a sequence that are separated by an arbitrary number of positions. More formally, LDDs are related to the rate of decay of statistical dependence of two points with increasing time interval or spatial distance between them. Commonly observed in natural languages, for example in English, there is a requirement for the subjects and verbs to agree, i.e., words bear relations and interact with other words in every sentence. LDDs are thus a pervasive feature of language which involve different faces such as agreement, binding, control and displacement among others [23], that imply a relation between two or more items. Such a relation valuate’s one item with respect to the other within a certain search space or domain, non-linearly but structurally defined [23]. Hauser et al. [24] show that natural languages go beyond purely local structure by including a capacity for recursive embeddings of phrases within phrases, which can lead to statistical regularities that are separated by an arbitrary number of words or phrases. Such long-distance, hierarchical relationships are found in all natural languages for which, at a minimum, a phrase-structure grammar is necessary [24]. Similarly, a mobility trajectory might display different degree of long-distance dependency depending on an individuals behavior, thus LDDs are challenging to model computationally. Typically, the predictability is directly dependent on the model’s ability to account for the LDDs present in a sequence.

### 2.4. Recurrent Neural Networks and Extensions

Several approaches have been used in an attempt to model the presence of LDDs in natural languages such as stochastic gradient decent [25]. RNN [26] have been proven efficient to model temporal data while accounting for the LDDs to a certain extent [27]. RNNs are a class of supervised machine learning models, comprised of artificial neurons with one or more feedback loops. The feedback loops are recurrent cycles over time or sequence which forms a hidden memory representation beneficial for processing and learning the dependencies between input sequences. A recurrent network is trained using a dataset consisting of a large number of input-target pairs with the objective to minimize the difference between the output and target pairs. This is performed by optimizing the weights of the network.

Modeling LDDs however is still challenging for simple/vanilla RNNs [28] due to the exploding and vanishing gradient (exponential decay of gradient, as it is back-propagated) problem [25]. Since RNN is a structure through time, the typical gradient decent is extended through time to train the network, called back-propagation through time (BPTT) [29]. However, computing error-derivatives through time is challenging, due to the unstable dynamics of RNN which renders gradient decent ineffective [30]. Thus, extensions to the vanilla-RNN were designed such as RNN-long short term memory (LSTM) [31] that enforce a constant error flow through the network thereby bridging the lags in the individual steps and thus addressing the above problem to some extent.

These extensions differ in their capacity to manipulate the internal memory and propagate gradients along the network [30]. More specifically, they differ with respect to the gating mechanisms employed [30], regularization techniques and the connections within the individual neurons and the hidden layers. We present a summary of the major architectural differences and the respective features associated with each extension selected in this work to carry of experiments in Table 2. We select the above models based on the contrasting nature of their connection architecture and the cell structure to quantify the contribution of such parameters on the modeling result. For instance, recurrent highway networks (RHNs) [32] are built to account for short and long-range correlations present in a sequence. On the other hand, pointer sentinel mixture models (PSMMs) [33] weigh long-range dependencies much higher than short-distance correlations in the sequence. In this paper, we quantify the performance of these variants to capture LDDs present in three datasets and analyze their applicability in various contexts.

### 2.5. Mutual Information

LDDs are challenging to detect and characterize due to a large number of associated parameters. Computing the mutual information of the data under consideration can be seen as a statical framework for discerning and quantifying the presence of LDDs and thus nature of the data generation process. Mutual information *I* is a quantity that measures the relationship between two random variables that are simultaneously sampled and quantifies the measure of information communicated, on average by one random variable about the other. *I* as a function of distance between the individual events can indicate the distribution of large but rare events and identify the presence of memory in the sequence. Mutual information, between two discrete random variables X,Y jointly distributed according to probability mass function p(x,y) is given by Equation (3).

(3) $$\begin{array}{cc} I(X;Y) & =\sum_{X,Y}p\left(X,Y\right)\log\frac{p(X,Y)}{p(X).p(Y)}\\ & =H(X)-H(X|Y)\\ & =H(Y)-H(Y|X)\\ & =H(X)+H(Y)-H(X,Y), \end{array}$$

where p(X,Y) is the joint distribution of two random variables *X* and *Y*, p(X) and p(Y) are the marginal distributions of *X* and *Y*. H(X,Y) is the joint entropy of two random variables, X,Y jointly distributed according to the pmfp(X,Y) and H(X|Y) is the conditional entropy of *X* given *Y*.

A related concept also used in our work is PMI. Unlike *I*, which quantifies the average information communicated by one symbol. In the context of mobility, a symbol refers to a point of interest. Thus, a sequence representing an individual’s trajectory is composed of temporally ordered points of interest. in the sequence about another, PMI quantifies the actual probability of co-occurrence of events p(X,Y) differing from the expectation. It is computed on the basis of the probabilities of the individual events under the assumption of independence p(X)p(Y) according to the Equation (4).

(4) $$PMI\left(X,Y\right)=log_{2}\frac{N.C(X,Y)}{C(X).C(Y)}.$$

PMI(X,Y)=0 indicates that *X* and *Y* are statistically independent. Here, C(X) and C(Y) is the total number of occurrences of *X* and *Y* respectively and C(X,Y) is the co-occurance of (X,Y). PMI is defined only over particular values of *X* and *Y*, and can therefore be negative, zero, or positive; it only considers the independence of those two particular values. A positive value of PMI indicates that the two events co-occur more frequently than would be expected under an independence assumption and a negative PMI means they cooccur less frequently than would be expected. Unlike PMI, I(X,Y) always takes non-negative values. I(X,Y)=m can be interpreted as the reduction in uncertainty about the event *Y* by *m* bits knowing the value of *X*.

Computing mutual information of a given dataset can quantify the presence of LDDs, leading to a suitable selection of predictive model a-priori. Pointwise Mutual information on the other had provides a fine-grained understanding of the dependencies within two symbols in a sequence. In this paper, we will rely on these measures to quantify LDDs present in the three mobility mobility datasets.

### 2.6. Entropy, Encoding and Compression

The seminal work of Shannon [36] defines entropy as the absolute minimum amount of storage and transmission required for capturing any information as opposed to raw data. Thus the entropy H(X) is equal to the amount of information learnt on an average from one instance of the random variable *X*. It is important to highlight that, the entropy does not depend on the value that the random variable takes, but only on the probability distribution p(x). The probabilities of different values can be leveraged to reduce the number of bits needed to represent the data if and only if the variable has non-uniform distribution. Thus, entropy can also be defined as the measure of compressibility of the data, or a measure that defines the predictability of a single random variable. Lower entropy therefore generally signifies higher predictability.

(5) $$H\left(X\right)=-\sum_{i}\left(p\left(i\right)\times log_{2}\left(p\left(i\right)\right)\right)=-E\left[\log\left(p\left(x\right)\right)\right].$$

Entropy rate on the other hand extends the concept of entropy from random variables to stochastic processes [37]. It is defined as the lower bound on the per-symbol description length when a process is losslessly encoded. In order to estimate the entropy rate of a stationary ergodic process, Kontoyiannis et al. [38] discuss a family of estimators and prove their point-wise and mean consistency. This approach runs a universal coding algorithm on the segment of the source output and averages the longest match-lengths. The resulting compression ratio can then be used as an upper bound for the entropy. If the segment length is long enough for the compression algorithm to converge, the compression ratio will be a good estimate for the source entropy. However it is important to note that there is no universal rate of compression [39].

Wyner et al. [40] performed an asymptotic analysis of the Lempel–Ziv algorithm [8] and found a relationship between the entropy rate and the asymptotic behavior of longest match-lengths. Building upon this relationship, Grassberger [41] suggested an entropy estimator based on average match-lengths for measuring the information of signals containing strong long-range correlations. Thus for any sequence $S_{N}$ of *N* binary digits, well-defined probabilities $P_{N}\left\{S_{N}\right\}$ exist for finding $S_{N}$ starting at any chosen site within *S*. Shannon’s entropy can also be written as $h=\lim_{N\to \infty }h_{N}$ where $h_{N}$ is defined as below:

(6) $$h_{N}=-\frac{1}{N}\sum_{S}p_{N}\left\{S_{N}\right\}log_{2}\times p_{N}\left\{S_{N}\right\}.$$

The quantities $h_{N}$ are called block entropies [42] where $h\leq h_{N}$ as the limit converges from the Shannon entropy equation. Grassberger [41] shows that these bounds are tight if the sequence has no long-range correlations. More precisely, $h_{n}=h$ for all n≥N if the sequence can be modeled by an $N^{th}$ order Markov chain. In this paper, we show that the currently used encoding schemes ignore the presence of dependencies in individual subsequences present in a mobility trajectory and thus result in an inflated entropy estimation and consequently in a deflated predictability bound.

### 2.7. Predictive Information

We now briefly discuss the predictive information theory, which provides a fine grained understanding of the interaction amongst the preceding concepts and they influence predictability. Predictive Information measures and quantifies how much of the past observations (can) tell us about the future [16]. The relationship between predictability, compressibility and temporally correlated entropy (time-series data) has been explored at length by Bialek et al. [16]. This is an important concept that ties the notions regarding event prediction, entropy and mutual information.

We restrict the discussion to sequential data, in which case predictive information diverges when the observed series allows to learn a more precise model of the data dynamics. Different variants of the predictions (next event, average event rate, event uncertainty etc.) are different slices through the conditional probability distribution. Greater concentration of this conditional distribution implies smaller entropy as compared to the prior distribution. The reduction in entropy can be viewed as the information that the past provides about the future [43]. Furthermore, in a time series if there is invariance under time translations, the entropy of the past data depends only on the duration of the observations [16]. The entropy of the sequence is thus in direct proportion to the observed duration and therefore the predictability is associated with the deviation of the entropy from extensivity. The average amount of information about the current state of a time-series is independent of how long the time-series has been observed. For models with a finite number of parameters, the stochastic complexity is proportional to the number of parameters and logarithmically dependent on the number of data points [43,44].

Finally we look at the result stated in Lin et al. [1]: mutual Information between two symbols, as a function of the number of symbols between the two, decays exponentially in any probabilistic regular grammar, but decays like a power law for a context-free grammar. This is an important observation relied upon in our work, given that human mobility has been known to follow context-free grammar [45]. Lin et al. [1] further state that exponential distribution is the only continuous distribution with the memory-less property. In order for a process to have a non-exponential probability distribution and satisfy the Markov property, the precise transition probability given the current state must be known. If a process has two or more states and transitions from each state with some non-exponential probability, then knowledge of the current state will not be sufficient to estimate the future distribution (next event prediction). It is important to note here that, for very short distances, power law decay and exponential decay are non-trivial to distinguish. Finally, Lin et al. [1] state that in a LDD driven system the number of bits of information provided by a symbol about another, drops as a power law with distance in sequences. This distance is defined as the number of symbols between the two symbols of interest. In this paper, we evaluate this observation made on natural languages on human mobility and use the results to verify the nature of human mobility.

## 3. Confirming $\pi^{max}$ Discrepancy with Real-World Datasets

In this section, we present the datasets used for all the empirical analysis conducted in this work and we formalize the notion of human mobility prediction. We discuss the accuracy results estimated using seven prediction techniques and compare them with respect to the theoretical upper bound. We confirm the discrepancy between $\pi_{acc}$ and $\pi^{max}$ and show that $\pi_{acc}⪕\pi^{max}$ does not always hold.

### 3.1. Experimental Setup

We now present the experimental setup for all the analysis performed in this work, starting with the description of the datasets used. We emphasize that the value of $\pi^{max}$ was dependent upon the experimental setup and dataset characteristics. Therefore it was essential to keep the same setup for computing $\pi^{max}$ and the empirical prediction accuracy for a legitimate comparison.

Real world Mobility Datasets. We conducted all the experiments by using three mobility datasets whose specifications are shown in Table 3. The PrivaMov dataset [46] was collected through GPS, WiFi and GSM in the city of Lyon (France) and includes university students, staff and their family members. The Nokia mobile dataset [47] (NMDC) was collected in the Lake Geneva region of Switzerland and consists young individuals’ trajectories, collected through GPS, WLAN, GSM and Bluetooth. The GeoLife dataset [13] was collected in Beijing (China) and contains trajectories recorded through GPS loggers and GPS-phones. Table 3 also presents the values (theoretical) of $S^{real}$ and $\pi^{max}$ computed using the approach mentioned by Song et al. [5] and Lu et al. [11] as per Equation (7) which is based on Lempel-Ziv data compression [8].

(7) $$S^{real}={\left(\frac{1}{n}\sum_{i=1}^{n}\lambda_{i}\right)}^{-1}log_{2}\left(n\right),$$

where *n* is the length of the trajectory (total number of locations) and λ is defined as the length of the shortest substring at an index *i* not appearing previously from index 1 to i−1. Note that we use the same base (2) in entropy estimation as for the logarithm in Fano’s inequality. Furthermore, the length of the substrings is set to zero upon reaching index *i*, when no more unique substrings can be computed using the above method. $\pi^{max}$ is then estimated by solving the limiting case of Fano’s inequality [48]. The computation of $S^{real}$ and $\pi^{max}$ at the aggregate level for the dataset was based on the independence of predictability on travel distance (radius of gyration $r_{g}$) in human mobility as demonstrated by previous studies [5,11,49].

Mobility prediction. We relied on the widely used definition of mobility prediction [10], which describes it as forecasting the transitions between places, after eliminating all self-transitions [4,12]. A preliminary step in achieving this consists of transforming the raw GPS locations into a sequences of points of interest [50]. A point of interest was defined as any location where an individual visits with an intentional purpose with a perceived priority for e.g., home/work place, gym, train station etc. Among the plethora of existing works dedicated to the problem of extracting these points, we rely on our approach that is independent of a priori assumptions regarding the data and individual mobility behaviors [50]. We then convert the raw GPS trajectory of a user *u*, $T_{u}=\langle \left(lat_{1},lon_{1},t_{1}\right),\left(lat_{2},lon_{2},t_{2}\right)\ldots \left(lat_{n},lon_{n},t_{n}\right)\rangle$, where $lat_{i},lon_{i}$ are the latitude and longitude coordinates respectively and $t_{i}$ is the timestamp such that $t_{i+1}>t_{i}$ into a sequence of temporally ordered points of interest, $s\left(t\right)=\langle \left(poi_{1},t_{1}\right),\left(poi_{2}t_{2}\right)\ldots \left(poi_{n},t_{n}\right)\rangle$, where $poi_{i}$ is the point of interest at index *i*. The mobility prediction task was thus formulated as: given a sequence s(t) up to a timestamp *n*, predict the next point of interest at timestamp n+1. The prediction accuracy was then estimated by following the approach stated by Lu et al. [11].

Predictive algorithms. We estimated the empirical predictability using seven different approaches: (1) Markov chains [10] (order 1-5), (2) hidden Markov model [51] (HMM), (3) vanilla recurrent neural network [34] (Vanilla-RNN), (4) recurrent neural network with long short-term memory [31] (RNN-LSTM), (5) dilated recurrent neural network [35] (Dilated-RNN), (6) recurrent highway network [32] (RHN), and (7) pointer sentinel mixture model [33] (PSMM). We use the standard implementations of the predictive algorithms as described in their respective papers. Markov chains [10] and hidden Markov models [51] are implemented using the standard python libraries (hmmlearn). We use hyper-parameters stated in these works (Table 4). Vanilla-RNN [34], RNN-LSTM [31] and dilated-RNN [35] are based on predicting the next character (language modeling) in the text, whereas RHN [32] and PSMM [33] model the prediction task as multivariate classification. For dilated-RNN [35] we used the dilations of 1, 2, 4, 8, 16, 32 and 64 and provided the results for dilation 32 after which we observed a drop in the accuracy.

Prediction accuracy. We computed the prediction accuracy ($\pi_{acc}$) of a dataset by estimating the average accuracy across all the individuals present in that dataset. Along the lines of [11], we measured the individual prediction accuracy by the proportion of accurate predictions over all days of that individual (users who were not active on a day are excluded in the prediction). The accuracy of a model is given by Equation (8).

(8) $$\pi_{acc}=\frac{\sum_{t=1}^{T}{𝟙}_{poi_{t}=poi_{t}^{*}}}{T},$$

where $poi_{t}$ is the true next point of interest of an individual at time *t*, $poi_{t}^{*}$ is the predicted next point of interest and *T* is the total number of prediction time-steps. The data is split into 10 windows consisting of 10% training set and the subsequent 10% as test set as performed in [5,11]. The training was performed in a cumulative manner such that the previous training instance was not lost. Such an approach also highlights the accuracy variations across the trajectory length in order to analyze the location dependencies and interaction distance.

### 3.2. Confirming the Predictability Upper Bound Discrepancy

**Figure 1:**
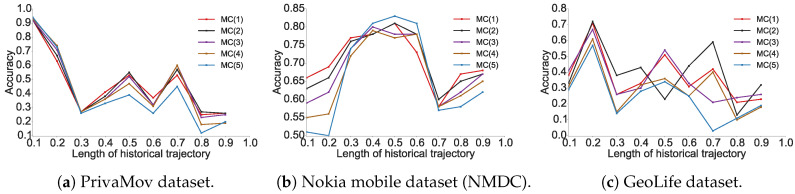
Prediction accuracy for Markov models (order 1–5). The x-axis signifies the proportion of trajectory length considered for the train-test split and y-axis signifies the precision of the prediction model.

**Figure 2:**
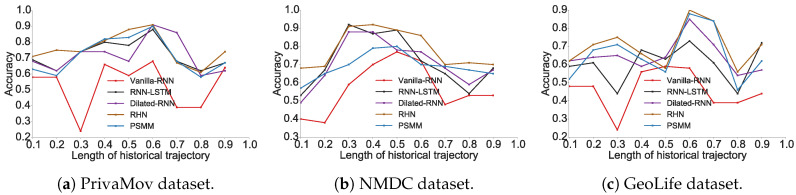
Prediction accuracy for recurrent-neural architectures. The x-axis signifies the proportion of trajectory length considered for the train-test split and y-axis signifies the precision of the prediction model.

We found that higher order Markov chains (typically >3) do not contribute to increased prediction accuracy, as also observed by Lu et al. [11]. The prediction accuracy for Markov chain models and the recurrent-neural architectures for all datasets is shown in Figure 1 and Figure 2, respectively. The experimental results show the proportion of accurate predictions for each day (in terms of dataset duration) based on the length of the historical trajectory accounted for to train the predictive model.

**Figure 3:**
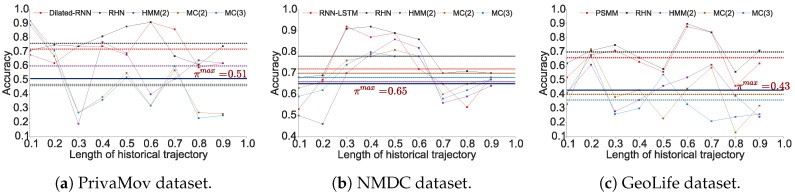
Comparison of $\pi^{max}$ with the maximum predictability achieved using models from each category. The dotted lines indicate the predictability by each approach (indicated with the same colour). x-axis signifies the proportion of trajectory length considered for the train-test split and y-axis signifies the precision of the prediction model.

We observed that the accuracy of Markov models ($\pi_{acc}$) lies in the vicinity of $\pi^{max}$. It was also clearly evident that recurrent-neural architectures significantly outperform Markov models with respect to their average accuracies. In Table 5, we show the maximum predictability achieved by using the best performing models from each algorithm, and in Figure 3 we compare their performance with the theoretical upper bound.

We also observed that in addition to the prediction model, the dataset characteristics significantly impacted the average accuracy. The average accuracy was the highest for the NMDC dataset [52], followed by PrivaMov dataset [53] and then GeoLife dataset [54]. We hypothesize that the accuracy values were governed by four key properties of mobility datasets namely; (a) number of unique points of interest, (b) average length of the trajectories, (c) number of interacting point of interests, and (d) the distance between these interactions. We systematically validated these assumptions in Section 4. We found that the NMDC dataset contains fewer points of interest, shorter average trajectory length (as shown in Table 3) and shorter interaction distance between the points as compared to the other two datasets (see Figure 12). Such short distance dependencies can be captured conveniently by Markov models of order 2, resulting in comparable accuracies to recurrent neural architectures. More importantly, variants of recurrent networks tend to show overfitting characteristics over datasets with smaller dependencies resulting in dropping accuracy on the validation set. This is the prime cause behind deep learning models showing poor performance against Markov models. We thus argue that precise quantification of dataset characteristics can guide towards selection of appropriate prediction models. The variation in the accuracy for a particular dataset with respect to the trajectory length under consideration stems from fluctuation of the interacting points and the distance within those interactions.

The prediction accuracies of recurrent-neural architectures also surpass the theoretical upper bound for the respective dataset. This anomaly in computing $\pi^{max}$ is puzzling, even more so considering the diversity of the datasets with respect to their collective time spans, visited number of locations, demographics and spatiotemporal granularity.

## 4. Revisiting the Underlying Assumptions

In this section we revisit the underlying assumptions listed in Section 1.3 involved in the upper bound derivation methodology and perform statistical tests to invalidate these assumptions.

### 4.1. Questioning the Markovian Nature of Human Mobility

Current mobility models [5,11] are based on the assumption that human mobility is memoryless or Poissonian. Such an assumption implies that consecutive events follow each other at relatively regular time intervals without the presence of very long waiting times. This Markovian assumption lies at the basis of the methodology used in deriving the upper bound for mobility predictability. The discrepancy between $\pi^{max}$ and $\pi_{acc}$ lets us questions the assumption that human mobility follows a Markov process. In this section, we conduct extensive analysis to validate the true nature of human mobility. More precisely we analyze the the distribution associated with several parameters of human mobility to check for slowly decaying, heavy-tailed processes.

In the following experiments, we check the power law fit using a Kolmogorov–Smirnov (K-S) statistic [55] based on the methodology adopted by Clauset et al. [56]. In order to estimate the likeliness of the data to be having drawn from the power law, we compute the *p* value and check its significance level. We also check the goodness of fit with other candidate distributions shown in Table 6 to exclude the possibility that no alternative distribution fits the data better than power law. We adopt the same approach for binned data as suggested by Virkar et al. [57]. The tests have been conducted using the powerlaw package powerlaw package: https://pypi.org/project/powerlaw/. The tests provide the log-likelihood ratio between the two candidate distributions *R*. This number will be positive if the data is more likely in the first distribution, and negative if the data is more likely in the second distribution. The significance value for that direction is *p*.

#### 4.1.1. Location Rank-Order Distribution

**Figure 4:**
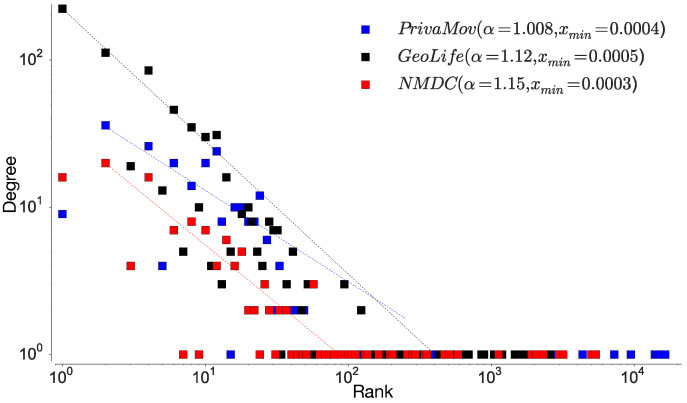
Rank distribution of location visits at the collective level for aggregated dataset. The data is binned into exponentially wider bins and normalised by the bin width. The straight line represents the fitting through least squares regression (α and $x_{min}$, computed through maximum likelihood estimation).

In order to gain insight into the datasets, we first analyze the rank distribution of the locations, according to the visit frequency at individual and aggregated levels. An individual visits different locations depending on a perceived priority attached to the location [15]; this results in a heterogenous location frequency distribution [18]. To study the location-rank distribution, we follow the approach stated in Zhao et al. [18] in order to rank locations according to their collective magnitude at the aggregate level. Figure 4 shows the rank distribution of visited locations in human mobility and Table 7 proves the existence of power law scaling (Zipf’s law [19]). We also observe a convergence and robustness at the individual level, which clearly indicates non-uniform mobility behavior and its effect on entropy, hinting at the non-Markovian nature of human mobility [18].

**Figure 5:**
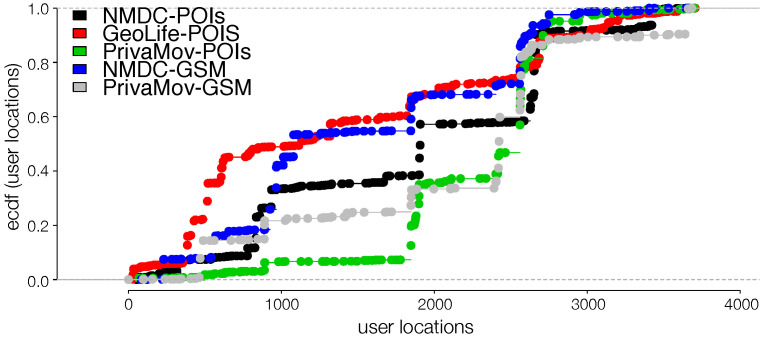
Empirical cumulative distribution for dataset points of interests and GSM logs.

Maximum likelihood estimation and Kolmogorov–Smirnov test. Maximum likelihood estimation is a tool for estimating the parameters as a data mining model. It is a computationally tractable way to learn a model from the data. Herein, we perform such fits according to [56]. Kolmogorov–Smirnov test (K-S test) is a non-parametric methodology that compares an observed distribution to, S(x) to a theoretical distribution F∗(x). In the above cases, the procedure consists of first forming the empirical cumulative distributions of S(x) (see Figure 5) and F∗(x) and estimating the difference between the candidate distribution fits (Table 8). The test is based on the following statistic:

(9) D=sup|F∗(x)−S(x)|,

with smaller values of D indicating a better fit to the corresponding theoretical distribution.

#### 4.1.2. Inter-Event Time Distribution

**Figure 6:**
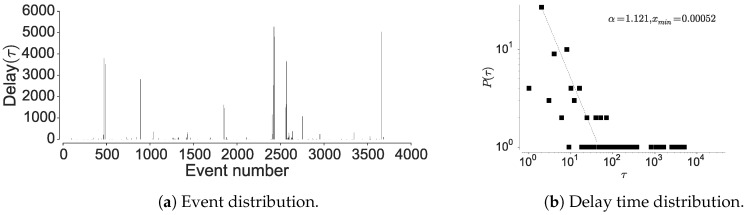
Distribution of the location visits and the delay between the visits in PrivaMov dataset.

**Figure 7:**
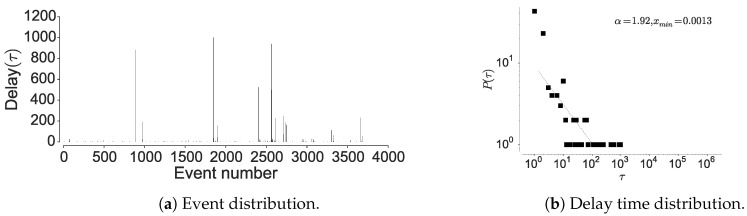
Distribution of the location visits and the delay between the visits in NMDC dataset.

**Figure 8:**
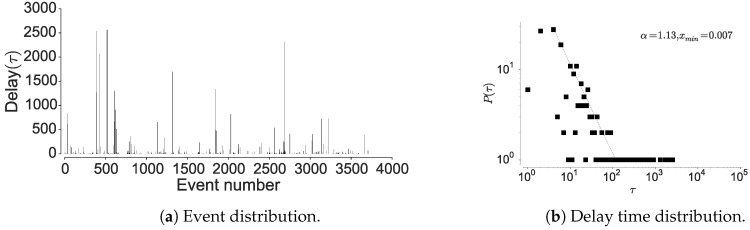
Distribution of the location visits and the delay between the visits in GeoLife dataset.

To further confirm the non-Markovian nature, we check the distribution of the inter-event times associated with the individual locations. Here, visiting a particular location is considered as an event and hence time between two location visits is considered as inter-event time. The current mobility models are based on an assumption that human movements are randomly distributed in space and time, hence are approximated by a Poisson process [15,19]. However, Barabasi [15] shows that human activities are non-Poissonian, by showing that inter-event timings depict long-tailed distribution. We observe a similar behavior when considering human mobility in all the datasets, when examining the inter-event and dwell times associated with each location; most locations are visited at high periodicity, while few locations encounter long waiting times. The current models assume that inter-event time follows exponential distribution [15], rather, we observe an emergence of power-law as seen in Figure 6, Figure 7 and Figure 8 corroborated by the statistical tests shown in Table 9. The spikes in the plot correspond to delays and display the visit regularity, which indicates a long-tailed process. The delay-time distribution depicts the priority list model in human mobility, bearing similarity to other activities as remarked by Barabasi [15]. When an individual is presented with multiple events under the context of mobility, the next location is determined on a perceived priority, thus resulting in power-law dynamics in inter-location waiting times [15]. This shows that the dwell-times associated with human mobility are not memoryless, hence cannot be considered as Markovian. In the above analysis, we also observe a convergence between individual mobility patterns and aggregated datasets, which concurs with the observations of Yan et al. [49].

#### 4.1.3. Mutual Information Decay

We validate Lin et at. [1,58] observation on mobility data where they state that; *I* as a function of the number of symbols (locations) between any two symbols and state that it would decay with a power-law for any context-free grammar and hence must be non-Markovian. With respect to human mobility trajectories, *I* between two location instances is the realization of a discrete stochastic process, with separation τ in time [1]. In order to analyze the existence of power law decay indicating the presence of memory in mobility trajectories we first consider the GeoLife [13] which is collected at a uniform sampling rate (location/5 s). We first validate the emergence of power law at distinct sampling rates by undersampling and oversampling the dataset by a factor of two and four. We perform oversampling by using semivariance interpolation [59]; a commonly used spatial interpolation scheme that fits the missing points by modeling the similarity between the points as a function of changing distance.

Mutual information between two location symbols is computed the estimating entropy of the marginal distribution of discrete random variables *X* and *Y*, and the joint entropy of discrete random variables *X* and *Y* as in Equation (10).

(10) $$I\left(X,Y\right)=H\left(X\right)+H\left(Y\right)-H\left(X,Y\right)=D_{KL}\left(p\left(XY\right)||p\left(X\right)p\left(Y\right)\right),$$

where *H*(*X*) is the entropy of a random variable *X* and *H*(*X, Y*) is the joint entropy of *X* and *Y*. $D_{KL}$ is the Kullback–Liebler divergence [60]. Thus, mutual information is same as the Kullback–Leibler divergence between distributions of *X* and *Y*. In order to compensate for insufficient samplings, we use the following adjustment proposed by Grassberger et al. [42] (Equation (11)) to compute *H*(*X*), *H*(*Y*), *H*(*X,Y*).

(11) $$H\left(X\right)=\log N-1/N\sum_{i=1}^{k}N_{i}\psi (\left(N_{i}\right).$$

Thus, we first estimate the distribution of *X* from index 0 followed by the distribution of *Y* at some index *d*, where the random variables *X* and *Y* are sampled from the individual trajectory sequence. *d* is then varied to compute long-distance dependencies at every separation by creating displacements between the random variables. Once the contextual dependence limit is reached, the process starts sampling noise, which sets the termination criterion and then the average similarity between the two symbols is quantified.

**Figure 9:**
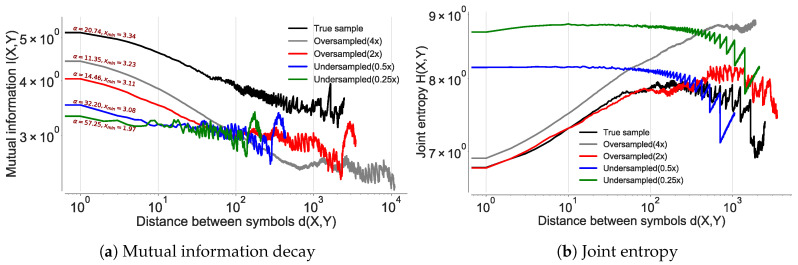
Mutual information decay for the GeoLife dataset at different sampling rates of the raw GPS coordinates projected onto a grid through Google S2 [61]. The upsampling was performed by the semivariance interpolation scheme [50].

As shown by Lin et al. [1], we observe a power-law decay at all the sampling rates (see Figure 9 and Table 10). This experiment validates the presence of LDDs in location sequences irrespective of their sampling rates. However, contrary to what would be expected that *I* would increase and decrease by the factor of under/over sampling, we observe a decrease in *I* for all the contexts in which the true distribution of the data is altered. We also observe that the reduction is proportional to the Kullback–Leibler divergence [60] between their respective distributions. The reduction in *I* stems from the fact that a change in the distribution results in the alteration of the true correlation between the location pairs. The true distribution will therefore show maximum *I*, compared to the cases when either artificial pairs are introduced (oversampling) or true pairs are removed (undersampling) from the dataset.

**Figure 10:**
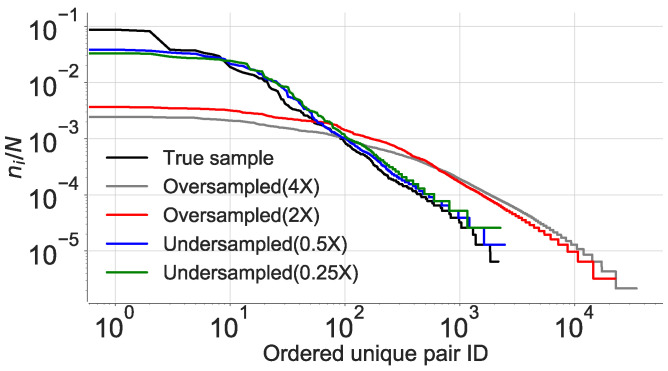
Location pair occurrences across all the sampling rates of the true sample. The x-axis represents the unique pair ID in the descending order of their frequency of occurrence. The y-axis is the ratio between the unique pairs and the total number of pairs contained in the an individual trajectory.

To verify our hypothesis, we calculate the joint entropy for all the cases and observe an increase in H(X,Y) for the altered distributions as shown in Figure 9b. We see that the increased entropy is due to an increase in the ratio between unique pairs in the dataset over the total number of pairs. The introduction of spurious pairs scrambles the true distribution as it leads to introduction of data points in the true sequence, thereby changing the random variables sampled at distance *d*, hence reducing *I*. This occurrence was confirmed after computing the area under the receiver operator characteristic (ROC), which was maximum for the true data distribution in the first quartile as compared to the rest as shown in Figure 10. This explains our observation of higher joint entropy for the oversampled and the undersampled case. This experiment also confirms that sampling rate of location coordinates would have a significant impact on the estimation of $\pi^{max}$ as also identified by [12].

**Figure 11:**
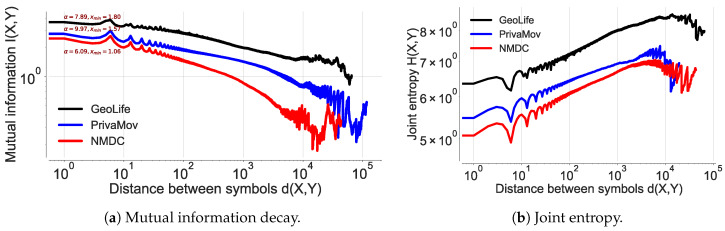
Mutual information decay and joint entropy estimated for all the datasets. The dataset consists of stacked sequences of temporally arranged individual points of interest.

After validating the existence of power law at different sampling rates, we analyze and quantify the presence of long-range correlations in other datasets. We observe a power law decay across all the datasets and their respective joint entropy, as shown in Figure 11a,b and corroborated by the statistical tests shown in Table 11. This information also serves as basis for the difference in accuracy for each dataset and the performance difference between the prediction algorithms. We further explore the Markov transition matrices for these datasets and observe that they are reducible and periodic, resulting in the decay of *I* to a constant. It has been shown that such a characteristic of the transition matrix cannot result in an exponential decay by Lin et al. [1,58]. This phenomenon is seen in a number of cases, including hidden and semi-Markov models [1,58].

In the literature, such behavior is superficially dealt with by increasing the state space to include symbols from the past, which does not address the main issue [58] with Markov models; lack of memory. This analysis shows that GeoLife dataset consists of considerably higher number of long-range correlations, compared to the PrivaMov dataset and the NMDC dataset. This should be self-evident from their respective data collection durations. However, the lower dependencies in the NMDC dataset, compared to PrivaMov, is due to the smaller area of the data collection region, which generally results in lower entropy of movement [5,11].

Here, we reason about the accuracy variation within and between the datasets and about the performance differences between the prediction algorithms. We observe that the NMDC dataset provides higher accuracy as compared to the other datasets, and witness a lower variation within the accuracies of different algorithms. This stems from the presence of very short dependencies in the individual trajectories present in the dataset, as seen in Figure 11a. The lower correlations also result in roughly equivalent prediction accuracies within the predictive models. The lower accuracies of recurrent-neural architectures, compared to Markov chain at some time-steps are due to the models tendency to actively seek for long-range dependencies. However, if the dataset does not contain such dependencies, the model underperforms, unless it is weighted to account for such an existence. This underperformance is evident from the behavior of dilated-RNN’s, where an increase in dilations (to account for longer dependencies) results in dropping accuracy. Such a phenomenon has also been observed in language modeling tasks, which suggests that this is not a domain specific occurrence [62]. The performance drop in the recurrent-neural architectures at different time steps is due to capturing the long-distance dependencies to different degrees, resulting in either under/over fitting. An additional reason for higher accuracy in NMDC dataset is due to a lower number of unique locations and smaller variations in the dwell-times, as compared to the PrivaMov and GeoLife datasets, as shown in Figure 4 and Figure 7. These aspects directly correlate with the entropy and thus affect predictability [5]. We also observe that PSMMs perform better on GeoLife dataset, compared to other two, due to its ability to search for dependencies at longer distances.

Our analysis of all the tests in this section, provides a compelling evidence that human mobility is characterized by a non-Markovian nature. More specifically, the presence of power law decay in these tests indicate a presence of memory which cannot be modeled by Markov processes. Furthermore, the diversity of the considered datasets with respect to the data collection region, duration, radius of gyration and sampling rate shows that this phenomenon is observable across disparate mobility behaviors. We thus invalidate the long held assumption that human mobility is Markovian by several prior works and confirm our first hypothesis which could have resulted in the inaccurate estimation of the predictability upper bound. In the next section, we analyze the impact of this assumption on the derivation of mobility entropy $S^{real}$ and consequently the predictability upper bound.

### 4.2. Questioning the Asymptotic Convergence of the Entropy Estimate

In this section, we investigate whether the entropy estimation schemes used in the current works provide an accurate characterization of the mobility entropy. Entropy estimation is the most crucial step towards computing the upper bound on mobility predictability using the Fano’s inequality [9,48]. We compare two significantly different variations of the Lempel–Ziv encoding algorithms with respect to their entropy estimates.

To this end, we check the scaling behavior of two variants of the Lempel–Ziv algorithm, the LZ78 [8] and the LZ77 scheme [63]. The current works [5,11,17,18] rely upon LZ78 data compression scheme [8] to compute the mobility entropy. The LZ78 scheme segments the complete trajectory sequence into substrings, where a substring is defined as the shortest subsequence in terms of its length, yet to be encountered. Song et al. [5] estimate entropy rate of an individuals trajectory according to Equation (7).

Theoretically, for a Markov process (of any order) Lempel–Ziv compression algorithms are optimal in achieving the compression limit put forth by Shannon and thus can be leveraged to estimate the entropy rate [21,64]. As it is non-trivial to estimate the entropy rate of information sources with strong long range correlations, we compare the two approaches with respect to their convergence. Assuming a binary sequence S=(1,0,1,1,0,1,0,1,1,0,1,1,0), LZ78 coding will break words $w_{1},w_{2}\ldots$ in a sequence *S* such that w1=s1 and $w_{k+1}$ is the shortest new word immediately following $w_{k}$. Thus *S* will be broken down into (1),(0),(11),(01),(011),(0110), here each word $w_{k}$ with k>1 is an extension of $w_{j}$ with j<k by one single symbol $s^{\prime }\in A$. LZ77 coding on the other hand, does not necessarily break $w_{k}$ as an extension of a previous word $w_{j}$, but can be an extension of any substring *S*, starting before $w_{k}$ and may even overlap it. Therefore, LZ77 will break down the sequence *S* as (1),(0),(11),(010),(11011).

The LZ77 scheme uses string-matching on a sliding window; whereas the second, LZ78, uses an adaptive dictionary. Furthermore, LZ77 coding does not necessarily break a substring as an extension of a previous subsequence and may therefore overlap it. Here, the average word length increases faster and the algorithm can make better use of long-range correlations. This stems from Grassberger’s [41] result, which states that as the block length increases more correlations are taken into account as a result of information/symbol decreasing with the number of elements in a block.

**Figure 12:**
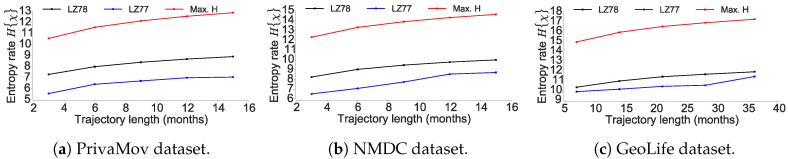
Comparison of entropy derived using LZ78 and LZ77 encoding algorithms. The red curve is the maximum entropy.

As seen in Figure 12, LZ77 clearly results in lower entropy as compared to the LZ78 scheme as observed by Storer et al. [65] who shows that LZ78 cannot truly capture long-range dependencies present in the sequence. One of the reasons for this as Schurmann [64] points out; LZ78 scheme based on shorter words is more efficient in the case of Bernoulli sources. However, in the case of the logistic map, the convergence of LZ77 scheme is faster then for the memoryless case. Thus, although LZ77 operates in the ignorance of the source statistics, it compresses the sequence better as compared to LZ78. However, we emphasize that it is still not the optimum scheme to compute the entropy as the information carriers of the sequence lie in its structural origin. We simply show that, the entropy measure provided by LZ78 scheme adopted by Song et al. [5] does not attain convergence. The maximum entropy here is computed by $log_{2}\left(k\right)$, where *k* is the cardinality of trajectory sequence. Grassberger [41] furthermore points out that LZ78 [8] and LZ77 [63] attain their claimed asymptotic behavior only when applied to Markov sequences. However, as previously established human mobility is not memoryless and therefore Markov property is not applicable in this case.

### 4.3. Questioning $S^{real}$ as a Relative Entropy Estimate for Human Mobility

Next, we inspect whether the current methodology ignores the presence of any long-range correlations present in the mobility sequence. In order to perform the above step, we compute the pointwise mutual information of an individual mobility trajectory. In order to analyze the long-distance dependencies between the elements of the individual substrings extracted by LZ78, we compute the PMI. This serves as a measure of the dependencies missed when the elements are grouped in distinct substrings as PMI computes the information provided by a symbol about another at a given distance *d*. Thus, we provide empirical evidence that the current entropy estimation scheme does not account for all the dependencies present in sequence.

**Figure 13:**
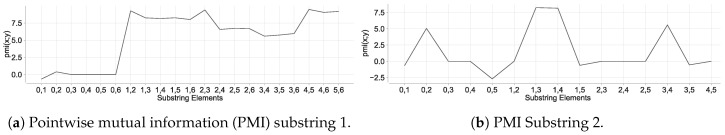
Pointwise mutual information across longer substrings in a user trajectory. The x-axis denotes the index’s of element pairs in a substring derived from a user trajectory using LZ78 encoding algorithm. The y-axis denote the pointwise mutual information between the element pairs.

**Figure 14:**
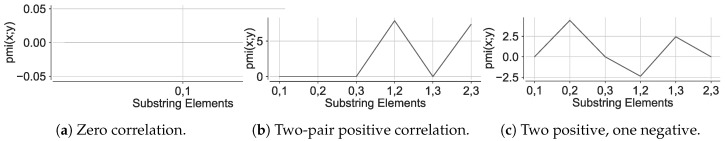
Pointwise mutual information across short substrings in a user trajectory. The x-axis denote the index’s of element pairs in a substring derived from a user trajectory using LZ78 encoding algorithm. The y-axis denote the pointwise mutual information between the element pairs.

We first see that a vast majority of substrings are of length one or two, which are dominant contributors to the entropy as also observed by Lesne et al. [66]. The estimated entropy is thus the outcome of finite-size fluctuations; and the total count of the substrings and of the elements in a substring does not represent the true probability distribution. As evident from Figure 13 the structural correlations between the elements of the individual substrings are ignored in case of long substring (number of elements > 5) but more surprisingly even in the case of short substrings (number of elements < 4) as seen from Figure 14. These correlations are ignored based on the argument that the probability of joint occurrences is weak [66]. This argument stems from the reasoning that the parsed substrings are independently and identically distributed according to Gaussian distribution, that does not apply to mobility trajectories. Finally, the correlated features can be compressed only by memorizing all the cases of intervening random variables between the correlated instances. [65]. It has thus been proved that Lempel-Ziv approach fails to capture redundancies in the data sources with long-range correlations [66].

Furthermore, as evident from Equation (7) the Lempel-Ziv approach limits the entropy estimation process at the sub-string level. Given that entropy is the complete quantitative measure of the dependency relations (including many point correlations), the computation of higher-order entropy is non-trivial. Therefore, it is flawed to assume that the $\pi^{max}$ derived from such an approximate estimation of $S^{real}$ should act as an upper bound of predictability on trajectories compiled for long time-spans. He shows that these bounds are tight if the sequence has no long-range correlations or more precisely, $h_{n}=h$ for all n≥N is the sequence is an $N^{th}$ order Markov chain. However, in case of mobility traces, these correlations are very strong (our experiments based on mutual-information (MI) decay verify this claim) and $h_{N}$ converges very slowly. We thus present an empirical evidence that the current approach used to estimate $S^{real}$ is not the true entropy associated with the mobility trajectory. But, it is in fact the entropy estimate derived by ignoring all the dependencies present within the individual elements of the substring extracted by the Lempel–Ziv encoding schemes. Ignoring the dependencies inflate the $S^{real}$ estimate and thus lowers the $\pi^{max}$

After estimating the mobility entropy $S^{real}$ as described in above, it is used to compute the predictability upper bound using Fano’s inequality [48]. We now present this notion to understand their inter-relation.

Consider estimating a random variable *X*, by an estimator $\hat{X}$ under the assumption that $P(\hat{X}\neq X)=\epsilon$. Joint entropy $H(X|\hat{X})$ is the average number of bits required to be transmitted in order to estimate *X* with the knowledge of $\hat{X}$. Fano’s inequality upper bounds this notion of estimating *X* given $\hat{X}$. Now, consider that we utilize some bits to communicate if *X* is $\hat{X}$ or not. The distribution for this is $P_{e},1-P_{e}$, i.e., we need to transmit $H(P_{e})$ bits on an average to successfully execute this task. If *X* is not $\hat{X}$, then it could be any one of the other |χ|−1 symbols in the alphabet (location point in the set of all possible locations). As a result the worst case length is log(|χ|−1) with a probability of $P_{e}$. Therefore Equation (12) quantifies this notion.

(12) $$H\left(X|\hat{X}\right)\leq H\left(P_{e}\right)+P_{e}.log\left(|\chi |-1\right)\leq H\left(P_{e}\right)+P_{e}.log\left(|X|\right).$$

Fano’s inequality, rooted in information theory [67], is intended for a data source with a well known probability distribution [48] which may not apply for mobility trajectories due to sampling, discretization and filtering schemes. Furthermore, the estimation of entropy by using Lempel–Ziv coding [8] was originally constructed to provide a complexity measure for finite sequences, i.e., input sequence displaying exponential decay in long-range correlations (memoryless structure). In this section, we thus demonstrate that when a mobility trajectory is further split in to smaller subsequences, the true distribution of the data is altered. This increases the associated entropy; and the derived $\pi^{max}$ thus acts as a limit on the Markov model.

## 5. Discussion

Even though large strides have been made in the field of human mobility modeling, much has to be done to understand the underlying dynamics of mobility behaviors. Through analyzing three large scale datasets containing mobility trajectories of individuals from several different countries, we still observe the discrepancy between $\pi^{max}$ and $\pi_{acc}$. Beneath this observation lie analysis for several assumptions made by previous works which has led to this inaccurate upper bound for mobility modeling. We have explored these assumptions from the lens of various information theoretic approaches such as mutual information, coding technics along with long-distance dependencies. In this section, we discuss the observations and provide a nuanced explanation of these observations.

Why do RNNs perform better? A key step in modeling mobility behavior is to interpret the characteristics of LDDs present in the mobility trajectories. As for a Markov process, the observations at $t_{n}$ depends only on events at previous time step $t_{n-1}$ or on previous *n* time-steps for an *n*-order Markov chain. Under such a context, the maximum possible predictive information is given by the entropy of the distribution of states at one time step, which is in turn bounded by the logarithm of the number of accessible states. Unlike Markov chains, the recurrent-neural architectures, such as RHN’s, approach this bound while maintaining the memory long enough so that the predictive information is reduced by the entropy of transition probabilities. Furthermore, the characteristics of LDDs depend on the number of interacting symbols and the distance between each interacting symbol, which is non-trivial to be modeled by a Markov process. In order to quantify LDDs, we use mutual information due to its simplicity and domain independence. As shown by Lin et al. [1], the mutual information decay offers some insights into why recurrent-neural architectures exceed probabilistic models in terms of capturing LDDs lying at multiple timescales. The ability of RNN’s to reproduce critical behavior stems from its architecture, where a long short-term memory (LSTM) cell will smoothly forget its past over a timescale of approximately $\log\left(1/f\right)\equiv \tau_{f}$. However, as described by [1] for timescales $\geq \tau_{f}$ the cells are weakly correlated and on timescales $\leq \tau_{f}$ the cells are strongly correlated. Therefore, a cell can remember its previous state for $\tau_{f}$ time steps and then grows exponentially with the depth of the network. At each successive layer, the gradient flow becomes exponentially sparse, which governs the growth of the forget timescale [1]. It has been recently shown that understanding the characteristics of LDDs can lead towards selection of better hyper-parameters for a model [68]. For instance understanding the scale of the dependencies can aid in selecting a suitable network depth, or the dilations of the dilated-RNN. In this work, we do not perform hyper-parameter tuning, which could have resulted in even higher estimates of $\pi^{max}$. Although estimation of a true upper bound is impractical, we hypothesize RNN models such as hierarchical-multiscale RNNs [20] could potential provide a very good $\pi^{max}$ estimates by capturing dependencies existing at several timescales.

Systematic bias. The wide range of the upper limit of mobility prediction in the previous works arise mainly due to the difference in the dependencies in their respective datasets collected for varying timespans. Other factors such as demographics, spatiotemporal resolution, radius of gyration, filtering and discretization schemes have a minor impact for longer duration datasets, typically exceeding three to four years. These factors gain importance in determining upper bounds and interpreting results of predictive performance for short duration datasets lasting one to two years. The previous research [5,11] estimated $S^{real}$ and $pi^{max}$ by using CDR datasets spanning a period of three to five months. Such datasets do not truly capture features such as the total number of unique locations visited by an individual, due to its low granularity (typically 4–5 km [17]). This results in a dataset with a masked entropy and mobility patterns ignoring long-range correlations. An important point to note is that for very short distances, power-law decay and exponential decay may not be trivial to differentiate [19]. This was due in part due to the fact that previous works [5,11] were only studied for short distances of human mobility and not due to unavailability of high granularity GPS datasets. Therefore, the assumptions underlying the computation of $S^{real}$ and $\pi^{max}$ would have been fairly easy to overlook.

Reinforcing this bias. The aforementioned inadequacies would reinforce the empirical validation of $\pi^{max}$ using Markov chains. However, as mentioned above, this would result in an error-prone estimation of predictability. As seen in other domains of sequential-data modeling such as natural language processing, Markov chains are fundamentally unsuitable for modeling such processes [23]. Our empirical observations, backed by theoretical foundations, indicate that human mobility will be poorly approximated by Markov chains. This is particularly true for trajectories that satisfy criteria of long time-span of collection.

Non-triviality of entropy estimation.It is non trivial to estimate the true entropy of mobility trajectory as the dependencies lie at several structural levels. Furthermore, the repeating patterns are typically hierarchical and they lie at various timescales. These scales depend on the mobility behaviors of the individual and therefore challenging to formulate a generic model. A more sophisticated description of these structures determining the mobility characteristics can be provided as more of the trajectory is observed. This results in an increase in the number of parameters in the model. That is, when we examine trajectories on the scale of individual coordinates, we learn about the rules of combining these points into points of interest and the transition paths between them. At the next level, if we consider several of these points of interest and the paths, we learn the rules for combining these points into semantic patterns. Similarly, when we look at semantic patterns, we learn about the visitation periodicities and circadian rhythms associated with the mobility behaviors. Therefore, longer traces have an increasing number of long range structural correlations that are non-trivial to be captured by the currently available entropy measure. One consequence of ignoring these structural properties is that the missed regularities are converted to apparent randomness. We empirically showcase this by computing the pointwise mutual information of the trajectories under consideration. We demonstrate that this problem arises particularly for small data sets; e.g., in settings where one has access only to short measurement sequences. Moreover, the current approximation implies that the substrings have the same compressibility factor [8], hence the results derived from this approach would coincide with the average. Thus, the current computation will result in higher estimates of entropy, consequently resulting in a lower predictability bound.

Effect of dataset characteristics on accuracy. As is clear from the accuracy charts that different datasets result in different accuracy values. Furthermore, we also observe variations in the average accuracy across the length of the trajectory. We highlight that determining the key characteristics of the dataset that affect the accuracy is not trivial. However, based on our experiments we find that the following factors indicate a correlation:

- number of unique locations present in the trajectory,
- length of the trajectory and the size of the dataset,
- number of interacting locations within a long-distance dependency,
- distance between the interacting locations.

We argue that precise quantification of the above characteristics could provide insights regarding the accuracy variations. More importantly the quantification of dataset characteristics can guide towards selection of appropriate prediction models.

All is not lost for Markov processes. Even though Markov models tend to underperform in modeling human mobility, their use in human mobility prediction is not entirely without interest. In fact, considering their low computational complexity, it might be advantageous to opt for a Markov model when a dataset contains short-distance dependencies and low number of unique locations. However, in datasets exhibiting LDD characteristics, long-range correlations appear in the vicinity of the system critical point, which can benefit from recurrent-neural architectures to accurately model human mobility. Therefore, quantifying the LDD characteristics of a dataset can aid in inferring where Markov models are applicable.

## 6. Conclusions

In this work, we scrutinized the methodology behind the upper bound estimation of human mobility prediction upon confirming the discrepancy of this limit with extensive experimentation. To this end, we revisited all the steps involved in the derivation of the upper bound. We first confirmed the discrepancy between $\pi_{acc}$ and $\pi^{max}$ by analyzing three mobility datasets and seven widely contrasting prediction models. We then systematically analyzed the assumptions underlying the derivation of $\pi^{max}$ and highlighted their shortcomings. We demonstrated the non-Markovian character in human mobility by conducting the statistical tests which confirmed the emergence of scaling laws in the distributions of dwelling times and inter-event times. We showed that mobility trajectories contain scale-invariant long-distance dependencies similar to natural languages unaccounted for by the upper bound computation methodology. We further quantified these dependencies measured by a power-law decay of mutual information and we claim that these assumptions culminate into the computation of an inflated entropy measure. We also showed that the exponent characterizing this decay is well defined for infinite sequences, however for mobility trajectories the accuracy of the analysis is restricted by the length of the substrings and their entropy. This explains why the empirical accuracy results surpass the theoretical upper bound in several previous research works and in our own experiments. Finally, we argued that the precise estimation of the predictability upper bound can be determined only when all the long-distance dependencies present in human mobility trajectories are accounted for by an entropy estimation scheme. However, we emphasized that usage of Markov models for modeling human mobility is still sometimes justified considering their low complexity for datasets containing short dependencies.

## Figures and Tables

**Table 1:** Comparison of $\pi^{max}$ and $\pi_{acc}$ at varying granularities of Δs (spatial granularity) and Δt (temporal granularity) reported by existing literature.

Authors (year)	$\pi^{\max}$ (Δs,Δt)	$\pi_{\mathrm{acc}}$	Prediction Model	Dataset Duration	Dataset Type
Song et al. [5] (2010)	93% (3–4 km)	–	–	3 months	CDR
Lu et al. [11] (2013)	88% (3–4 km)	91%	Markov (first-order)	five months	CDR
Smith et al. [12] (2014)	93.05–94.7% (350 m, 5 min) 81.45–85.57% (100 m, 5 min) 74.23–78.20% (350 m, 60 min)	–	–	36 months	GPS
Ikanovic and Mollgaard [17] (2017)	95.5 ± 1.8% (1.7 km) 71.1% (25 m)	88.3 ± 3.8% 75.8%	Markov (first-order)	36 months (same as previous)	GPS

**Table 2:** Recurrent neural network variants with their respective architectural differences and features.

Extension	Architecture	Features
Vanilla-RNN [34]	• no cell state/gating mechanism • recurrent connections	• faster and stable training • simple architecture
RNN-LSTM [31]	• similar connections as Vanilla-RNN • diff. cell state with gating mechanism	• actively maintain self-connecting loops • prevents memory degradation
Dilated-RNN [35]	• similar cell structure as LSTM • dilated skip connections	• increased parallelism in the computation • improves long-term memorization capabilities
RHN [32]	• diff. cell design • long credit assignment paths	• handles short-term patterns • reduces data-dependent parameters for LDD memorization
PSMM [33]	• diff. gating function, shortcut connections • variable dimensionality hidden state	• improves handling of rare symbols • allows for better long-distance gradients

**Table 3:** Mobility dataset specifications and their respective $S^{real}$ and $\pi^{max}$ values.

Datasets	Num. Users	Duration (months)	Avg. Trajectory Length	Distinct Locations	Avg. Spatio-Temporal Granularity	$S^{\mathrm{real}}$	$\pi^{\max}$
**PrivaMov**	100	15	1,560,000	2651	246 m 24 s	6.63	0.5049
**NMDC**	191	24	685,510	2087	1874 m 1304 s	5.08	0.6522
**GeoLife**	182	36	8,227,800	3892	7.5 m 5 s	7.77	0.4319

**Table 4:** Hyperparameters selected for each recurrent neural networks (RNN) variant for the prediction accuracy measurement experiments.

RNN Variant	Hidden-Layer Size	Unroll Steps	Learning Rate	Activation Function	Optimizer	Dropout Rate
Vanilla-RNN	100	25	0.1	tanh	Adam	0.2
RNN-LSTM	100	50	1.0 × ${}^{-8}$	ReLU	Adam	0.2
Dilated-RNN	100	32	1.0 × ${}^{-6}$	ReLU	Adam	0.2
RHN	100	50	1.0 × ${}^{-8}$	ReLU	Adam	0.2
PSMM	100	50	1.0 × ${}^{-8}$	ReLU	Adam	0.2

**Table 5:** Prediction accuracy achieved using the best performing models for each dataset.

Datasets	$\pi^{\max}$	$\pi_{\mathrm{acc}}\left(\mathrm{MC}\left(2\right)\right)$	$\pi_{\mathrm{acc}}\left(\mathrm{MC}\left(3\right)\right)$	$\pi_{\mathrm{acc}}\left(\mathrm{HMM}\left(2\right)\right)$	$\pi_{\mathrm{acc}}\left(\mathrm{RHN}\right)$	$\pi_{\mathrm{acc}}\left(\mathrm{RNN}\right)$
**PrivaMov**	0.50	0.47	0.46	0.60	0.76	0.72 (Dilated-RNN)
**NMDC**	0.65	0.70	0.68	0.66	0.78	0.72 (RNN-LSTM)
**GeoLife**	0.43	0.40	0.36	0.43	0.70	0.66 (PSMM)

**Table 6:** Candidate distributions used for assessing the power law fit to the statistical tests.

Name	Density p(x)=Cf(x)	
	f(x)	C
Power law with cutoff	$x^{-\alpha }e^{-\lambda x}$	$\frac{\lambda^{1-\alpha }}{\tau (1-\alpha ,\lambda x_{min})}$
Exponential	$e^{-\lambda x}$	$\lambda e^{\lambda x_{min}}$
Stretched exponential	$x^{\beta -1}e^{-\lambda x^{\beta }}$	$\beta \lambda e^{\lambda x_{min}^{\beta }}$
Log-normal	$\frac{1}{x}exp\left[-\frac{{\left(lnx-\mu \right)}^{2}}{2\sigma^{2}}\right]$	$\sqrt{\frac{2}{\pi \sigma^{2}}}{\left[erfc\left(\frac{lnx_{min}-\mu }{\sqrt{2}\sigma }\right)\right]}^{-1}$

**Table 7:** Kolmogorov–Smirnov goodness-of-fit test for location rank-order distribution.

Rank Order	Power Law p	Log-Normal		Exponential		Stretched Exp.		Power Law + Cutoff		Support for Power-Law
		LR	p	LR	p	LR	p	LR	p	
Privamov	0.00	−12.72	0.00	−30.12	0.00	−11.42	0.00	−113.1	0.00	with Cutoff
NMDC	0.00	−11.28	0.00	−27.23	0.00	−13.95	0.00	−320	0.00	with Cutoff
Geolife	0.006	−17.04	0.00	−19.21	0.00	−18.21	0.08	−560.78	0.00	with Cutoff

**Table 8:** Maximum likelihood and K-S test for the cumulative distributions (lower value in boldface indicates a better fit). We clearly observe that high granularity points of interest depict a power-law unlike the CDR logs which are a rough approximation of human mobility.

MSE				
Measure	Log-Normal	Exponential	Stretched Exp.	Power Law + Cutoff
**NMDC-POIs**	0.04501	0.05648	0.02348	**0.00616**
**GeoLife-POIs**	0.00324	0.07306	0.00378	**0.00087**
**PrivaMov-POIs**	0.05824	0.09386	0.00739	**0.00114**
**NMDC-GSM**	0.25584	**0.00224**	0.00584	0.07268
**PrivaMov-GSM**	0.03655	0.00895	**0.00098**	0.00783
**K-S Test**				
**NMDC-POIs**	0.65843	0.75615	0.07456	**0.00825**
**GeoLife-POIs**	0.63288	0.93644	0.04289	**0.00046**
**PrivaMov-POIs**	0.96752	0.69748	0.27896	**0.00116**
**NMDC-GSM**	0.56825	0.00987	**0.00967**	0.04568
**PrivaMov-GSM**	0.85621	0.00567	**0.00165**	0.00927

**Table 9:** Kolmogorov–Smirnov goodness-of-fit test for inter-event time distribution.

Inter-Event Times	Power Law p	Log-Normal		Exponential		Stretched Exp.		Power Law + Cutoff		Support for Power-Law
		LR	p	LR	p	LR	p	LR	p	
Privamov	0.12	−1.13	0.28	5.69	0.00	0.09	0.00	−0.34	0.74	with Cutoff
NMDC	0.08	−0.11	0.02	2.98	0.00	3.78	0.54	−2.87	0.31	weak
Geolife	0.86	−7.76	0.00	−20.43	0.00	17.87	0.08	−0.30	0.59	good

**Table 10:** Kolmogorov–Smirnov goodness-of-fit test for mutual information decay of GeoLife dataset at varying sampling rates.

Sampling Rate	Power Law p	Power Law + Cutoff		Log-Normal		Exponential		Stretched Exp.		Support for Power Law
		LR	p	LR	p	LR	p	LR	p	
1X	0.51	5.43	0.19	0.278	0.47	9.89	0.96	4.32	0.12	good
2X	0.06	0.00	0.07	−1.25	0.08	2.89	0.11	10.08	0.00	with Cutoff
4X	0.46	−0.065	0.67	−0.072	0.87	1.89	0.87	1.78	0.07	moderate
0.5X	0.00	0.00	0.00	−5.54	0.01	8.66	0.38	11.88	0.00	with Cutoff
0.25X	0.00	0.00	0.02	−1.78	0.03	9.94	0.04	13.56	0.00	with Cutoff

**Table 11:** Kolmogorov–Smirnov goodness-of-fit test for mutual information decay across all the datasets.

Dataset	Power Law p	Power Law + Cutoff		Log-Normal		Exponential		Stretched Exp.		Support for Power Law
		LR	p	LR	p	LR	p	LR	p	
**Privamov**	0.43	3.25	0.69	1.78	0.28	6.28	0.83	4.89	0.34	good
**NMDC**	0.27	1.82	0.11	−0.27	0.10	2.47	0.65	2.21	0.16	moderate
**Geolife**	0.51	5.43	0.19	0.278	0.47	9.89	0.96	4.32	0.12	good

## Data Availability

GeoLife dataset that supports the findings of this study is public and is made available by Microsoft Asia [54]. The PrivaMov dataset is collected by Universite de Lyon and can be obtained by submitting an online form [53]. The Nokia Mobile dataset (NMDC) can be obtained in a similar fashion [52]. Furthermore, our source codes are made public [69] and further clarifications will be provided upon request.
